# Development and application of crude sap-based recombinase polymerase amplification assay for the detection and occurrence of grapevine geminivirus A in Indian grapevine cultivars

**DOI:** 10.3389/fpls.2023.1151471

**Published:** 2023-03-09

**Authors:** Gopi Kishan, Rakesh Kumar, Susheel Kumar Sharma, Nishant Srivastava, Nitika Gupta, Ashwini Kumar, Virendra Kumar Baranwal

**Affiliations:** ^1^ Advanced Centre for Plant Virology, Division of Plant Pathology, ICAR-Indian Agricultural Research Institute, New Delhi, India; ^2^ ICAR-Indian Institute of Seed Science, Kushmaur, Mau, Uttar Pradesh, India

**Keywords:** RPA, grapevine, geminiviruses, detection, crude sap

## Abstract

Geminiviruses are known to infect several fields and horticultural crops around the globe. Grapevine geminivirus A (GGVA) was reported in the United States in 2017, and since then, it has been reported in several countries. The complete genome recovered through high-throughput sequencing (HTS)-based virome analysis in Indian grapevine cultivars had all of the six open reading frames (ORFs) and a conserved nonanucleotide sequence 5′-TAATATTAC-3′ similar to all other geminiviruses. Recombinase polymerase amplification (RPA), an isothermal amplification technique, was developed for the detection of GGVA in grapevine samples employing crude sap lysed in 0.5 M NaOH solution and compared with purified DNA/cDNA as a template. One of the key advantages of this assay is that it does not require any purification or isolation of the viral DNA and can be performed in a wide range of temperatures (18°C–46°C) and periods (10–40 min), which makes it a rapid and cost-effective method for the detection of GGVA in grapevine. The developed assay has a sensitivity up to 0.1 fg μl^-1^ using crude plant sap as a template and detected GGVA in several grapevine cultivars of a major grapevine-growing area. Because of its simplicity and rapidity, it can be replicated for other DNA viruses infecting grapevine and will be a very useful technique for certification and surveillance in different grapevine-growing regions of the country.

## Introduction

Grape is one of the first and important fruit crops to be cultivated by man (McGovern, 2019). Viruses are among the major biotic stressors for grapevine, and more than 80 viruses and other graft-transmissible agents like viroids have been reported so far (Fuchs, 2020). Most of these are RNA viruses that include leafroll disease-causing ampeloviruses [grapevine leafroll-associated virus 1 (GLRaV-1), GLRaV-3, GLRaV-4, and GLRaV-7], closterovirus (GLRaV-2), Shiraz disease-causing vitiviruses [grapevine virus A (GVA), grapevine virus B (GVB)], and stem pitting-associated foveavirus [grapevine rupestris stem pitting-associated virus (GRSPaV)] (Martelli, 2014).

Several DNA viruses such as grapevine red blotch-associated virus [*Grablovirus* (GRBV)] (Krenz et al., 2012; Al Rwahnih et al., 2013), grapevine vein clearing virus (*Badnavirus*) (Zhang et al., 2011; Qiu and Schoelz, 2017), grapevine Roditis leaf discoloration-associated virus (*Badnavirus*) (Maliogka et al., 2015), and grapevine geminivirus A (GGVA) (*Maldovirus*) have also been reported to infect grapevine (Al Rwahnih et al., 2017). These DNA viruses alter the development of berries and consequently negatively impact yield (Sudarshana et al., 2015; Blanco-Ulate et al., 2017). Geminiviruses are monopartite or bipartite having circular single-stranded DNA (ssDNA) genome infecting both herbaceous and woody plants. Fourteen different genera, namely, *Becurtovirus*, *Begomovirus*, *Capulavirus*, *Citlodavirus*, *Curtovirus*, *Eragrovirus*, GRBV, *Maldovirus*, *Mastrevirus*, *Mulcrilevirus*, *Opunvirus*, *Topilevirus*, *Topocuvirus*, and *Turncurtovirus*, are included in the *Geminiviridae* family (Fiallo-Olivé et al., 2021 **).** GGVA is assigned to *Maldovirus* that also includes apple geminivirus 1 and Juncus maritimus geminivirus 1. All of the maldoviruses have a monopartite genome and contain a conserved nonanucleotide sequence (5′-TAATATTAC-3′), a characteristic feature of all of the geminiviruses (Sun et al., 2020; Fiallo-Olivé et al., 2021). GGVA was reported by Al Rwahnih et al. (2017) for the first time in 2017 from the United States during screening of two table grape accessions received from South Korea. In recent years, it has been reported in other parts of the world such as China (Fan et al., 2017; Sun et al., 2020), South Korea (Jo et al., 2017), and New Zealand (Veerakone et al., 2019). From India, its presence was reported through bioinformatic analysis of publicly available raw sequence reads of the grapevine cultivar ‘Red Globe’ (Sidharthan et al., 2020). GGVA contains an ssDNA genome (size of 2,903–2,907 nucleotides) having six open reading frames (ORFs) and a conserved ′TAATATTAC′ sequence and is monopartite in nature (Sun et al., 2020).

Detection of grapevine viruses becomes crucial to control their spread and reduce the subsequent impact on quality and yield. Several molecular (RT-PCR, q-PCR) and next-generation sequencing (NGS)-based techniques have been employed to efficiently detect these viruses (Sidharthan et al., 2022). However, isothermal amplification-based detection assays such as recombinase polymerase amplification (RPA) and loop-mediated isothermal amplification (LAMP) are becoming popular for targeted detection due to their sensitivity, specificity, and attribute of not requiring stringent thermal cycling (Sharma et al., 2023). RPA is based on the utilization of three different enzymes, viz., a recombinase, an ssDNA-binding protein (SSB), and a strand-displacing polymerase (Piepenburg et al., 2006). RPA reactions require an isothermal temperature of 25°C–42°C and can be completed within 5–30 min depending on the concentration of nucleic acid and size of the amplicon. RPA in general has advantages over the parallel nucleic acid-based detection methods in terms of its applicability in an isothermal temperature, short reaction time, and robustness. The RPA assay has been employed to detect both DNA [*Nanoviridae* (Kapoor et al., 2017; Cao et al., 2020); *Caulimoviridae* (Kumar et al., 2018; Mohandas and Bhat, 2020); *Geminiviridae* (Londoño et al., 2016)] and RNA [*Closteroviridae* (Mekuria et al., 2014; Sharma et al., 2023); *Potyviridae* (Zhang et al., 2014; Kumar et al., 2021b); *Fimoviridae* (Babu et al., 2017); *Bromoviridae* (Srivastava et al., 2019); *Alphaflexiviridae* (Kumar et al., 2021a); *Betaflexiviridae* (Kumar et al., 2022)] viruses of several field and horticultural crops. Grapevine viruses such as GRBV (through AmplifyRP^®^ Acceler8 assay) and GLRaV-3 (through AmplifyRP^®^ XRT assay) have also been detected successfully using RPA-based assays (Li et al., 2017). In grapevine, purified nucleic acid has been used as a template for RPA-based detection of GRBV and GLRaV-3 (Li et al., 2017). With this background information, the present study was designed to develop a fast, robust, sensitive, and feasible detection assay that requires minimum laboratory setup and can also be operated at field level using crude sap for efficient detection of GGVA in large-scale grapevine samples and planting material.

## Materials and methods

### Plant sample

Virome analysis of grapevine cultivars ‘Super Sonaka’ and ‘Anushka’ (clonal selection of Thompson Seedless) was performed using robust high-throughput sequencing (HTS) coupled with bioinformatic tools. High-quality ribo-depleted RNA was used for library preparation, and sequencing was done using Illumina NovaSeq 6000 V1.5 sequencing system (Illumina, San Diego, CA, USA). Furthermore, employing *de novo* assembly and homology search based on NCBI Non Human Host Virus sequences, a multitude of viruses has been identified along with GGVA (unpublished data). These plants were maintained in the containment facility of Indian Agricultural Research Institute (IARI), New Delhi, and used for optimization of RPA. The complete genome of GGVA (2,905 nt) recovered from ‘Anushka’ is submitted to NCBI Gene Bank (Accession No. OQ079131 and OQ079132). The phylogenetic tree of these recovered sequences with other available world genomes of GGVA was constructed using the maximum likelihood (ML) method and Kimura 2-parameter model in MEGA11 with 1,000 bootstrap replicates. PCR-based wet-lab confirmation was done employing coat protein (CP)-based primer pair (GGVA-Fp579 5′–3′: CGCAGGTCAAGTCAGTCAGT and GGVA-Rp 579 5′–3′: TTTTGCACCTGCATCCGAAC), amplifying the 579-bp genomic region. Symptomatic plants showing leaf yellowing and chlorosis from ‘Super Sonaka’, ‘Anushka’, and ‘Manik Chaman’, common table grape fruit cultivars, were tested for GGVA using already available PCR primers before using them for the RPA assay. Field samples were collected from grapevine-breeding plots (10 different cultivars) of ICAR-IARI, New Delhi, and 30 vineyards (representing five grapevine cultivars) from a major table grape production area in Anantapur district of Andhra Pradesh, India. Five plants were randomly selected from individual vineyards during sample collection from the Anantapur region and pooled to make one sample. Pooling was done to elucidate the GGVA infection status of a particular vineyard and to make the assay cost-effective. Two PCR-negative plants were used as the control for subsequent experiments conducted during the course of the study.

### RNA and DNA isolation

Purified RNA and DNA were isolated from petioles and midrib regions of collected leaves of three grapevine cultivars (Super Sonaka, Anushka, and Manik Chaman), and both were used as a template for the detection of GGVA. RNA isolation was done using Spectrum™ Plant Total RNA kit (Sigma-Aldrich, St. Louis, MO, USA) with slight modifications by adding 2% polyvinylpyrrolidone to the lysis solution at the time of sample crushing. DNA extraction was carried out according to the standard cetyltrimethylammonium bromide (CTAB) method and also using DNeasy^®^ Plant Mini Kit (QIAGEN GmbH, Hilden, Germany). Extracted RNA/DNA from each sample was eluted in 40 µl of nuclease-free water. The quantity and quality of the extracted RNA and DNA were assessed using NanoDrop One Spectrophotometer (Thermo Fisher Scientific, Waltham, MA, USA) and electrophoresis in 1% agarose gel.

### Crude extract preparation

Leaf samples were processed to extract DNA for the RPA-based detection assay using different extraction buffers, viz., 0.5 M NaOH solution (Kapoor et al., 2017), phosphate buffer pH 7.4, NaOH : EDTA (1:1) (Sharma et al., 2023), and lysis buffer from DNeasy^®^ Plant Mini Kit (QIAGEN GmbH, Hilden, Germany). In brief, 100 mg of leaf tissues from the petiole and midrib portion was ground in the extraction buffers (1:10 w/v) using sterile mortar and pestle. The crude sap was transferred to a 2-ml microcentrifuge tube and centrifuged for 3 min at 12,000 rpm. The supernatant was collected without disturbing the particulate matter formed at the bottom of the tube and transferred to a sterile 2-ml collection tube for use as a template for the RPA-based detection assay.

### Primer designing

PCR-positive samples (Super Sonaka, Anushka, and Manik Chaman) for the CP genomic region-based primers (GGVA-Fp 579 and GGVA-Rp 579) were used for all subsequent RPA assays. The RPA primer pair was designed following the guidelines of the manufacturers (www.twistdx.co.uk). GGVA complete genomes recovered through HTS (OQ079131 and OQ079132) along with other available world genomes (KX517616, MZ488502, MF163262, MK690474, KX570618, KX570617, MF163264, KX950822, and NC031340) were used for primer designing (www.ncbi.nlm.nih.gov). The RPA primer pair GGVA-RPA212-Fp (CTACCTATGTATCTATGCCTCATTTGGG) and GGVA-RPA212-Rp (CCCTCCACCAGTAAACAGATCATAAAAG) was selected from the CP conserved region after aligning all of these sequences through CLUSTALW multiple alignment (Bio-Edit). The specificity of the primers was assessed by *in silico* analysis using BLASTn (http://www.ncbi.nlm.nih.govt/blast). These primers were 28 nt long and amplified amplicons of 212 bp.

### Primer validation through the PCR assay

The designed primer pair was validated using a standard PCR assay. The isolated DNA and cDNA (prepared from RNA) from positive samples were used as template, and a reaction mixture of 20 µl was prepared. The PCR program of initial denaturation at 94°C for 5 min followed by 35 cycles of denaturation at 94°C for 30 s, annealing at 56°C for 40 s, extension at 72°C for 35 s, and a final extension at 72°C for 10 min was performed. The resulting PCR amplicons were separated on 1.0% agarose gel at 85 V, and a 100-bp ladder (GeneDireX^®^) was used for product size estimation through visualization in a gel documentation system (Bio-Rad, Gel Doc XR system) under UV light. Furthermore, the DNA products from gel slices were eluted using NucleoSpin^®^ Gel and PCR Clean-Up Kit (Macherey-Nagel, Duren, Germany) as per manufacturer’s protocol. Cloning of eluted products was done using pGEMT vector, and sequencing of the cloned products was carried out in paired-end manner at Barcode Biosciences (Bengaluru, Karnataka, India). The sequences obtained were further aligned using BLASTn to verify the virus and submitted to the NCBI database.

### Recombinase polymerase amplification assay using DNA, RNA, and crude sap as template

The RPA assay was performed as per manufacturer’s guidelines using TwistAmp^®^ Basic kit (TwistAmp^®^ Basic-TwistDx limited). In brief, a reaction mixture of 45 µl was prepared by adding forward primer: 2.4 µl (10 µmol µl^-1^), reverse primer: 2.4 µl (10 µmol µl^-1^), rehydration buffer: 29.5 µl, and sterile distilled water of 10.7 µl. Freeze-dried pellet was added to this mixture and mixed thoroughly. Equal volumes (22.50 µl) of this mixture were distributed into two PCR tubes, and template (DNA, RNA, and crude sap lysed in 0.5 M NaOH solution) of 1 µl was added. At the end, 1.50 µl of magnesium acetate (280 mmol) was added to start the reaction and incubated at 38°C for 30 min for amplification. For the reaction in which RNA was used as template, 1 µl of reverse transcriptase was also added. The reaction was heat-inactivated at 65°C for 10 min, and the product was loaded on 1.5% agarose gel. Also, 5% Sodium Dodecyl Sulfate (SDS) (w/v) was added to the final mixture to observe the differences in band formation in comparison to those of the heat-inactivated one.

### Comparison of incubation temperatures and time limit for efficient detection of grapevine geminivirus A (GGVA) through the RPA assay

The RPA assay was performed at different incubation temperatures of 10°C, 14°C, 18°C, 22°C, 26°C, 30°C, 34°C, 38°C, 42°C, and 46°C to ascertain the limit and the most suitable temperature to perform the reaction. The reactions were kept for 30 min at these different temperatures using crude plant extract (lysed in 0.5 M NaOH solution) from the positive sample as a template. Similarly, to know the limit and best period required for obtaining confirmative results, the RPA reaction was kept for different periods, viz., 10, 15, 20, 25, 30, 35, and 40 min at an isothermal temperature of 38°C.

### Sensitivity and specificity comparison of the RPA assay with PCR

Detection limit or sensitivity comparison of RPA and conventional PCR was carried out using different templates, viz., DNA, RNA, plasmid DNA (GGVA viral insert cloned in pGEMT vector), and crude sap obtained from a GGVA-positive sample. The plant sample was found negative in the RPA, and PCR was used as healthy control. For obtaining different dilutions of templates, a 10-fold serial dilution technique was followed. In this, extracted templates having an initial concentration of 100 ng/µl were used and considered as 10^0^. For DNA, 1 µl from 10^0^ was taken and added to 9 µl of DNA extracted from healthy plants and serially diluted up to the strength of 10^-10^. The same procedure was followed for other templates, and 1 µl of an aliquot from 10^0^ obtained from the positive sample was added to 9 µl of the respective templates extracted from healthy plants. For plasmid DNA as template, initial stock (100 ng/µl) was considered as 10^0^ from which 1 µl was taken and mixed with 90 µl of healthy crude sap in a new tube to obtain 10^−1^ and serially diluted up to the strength of 10^-15^. In this study, 1 µl of an aliquot from all of these dilutions was used as template in their respective reaction mixtures prepared for both RPA and PCR reactions. The RPA reactions for all of the sensitivity analysis experiments were performed at an isothermal temperature of 38°C for 30 min.

To validate the specificity of RPA primers designed for the detection of GGVA through the RPA assay, different grapevine samples found to be positive for different viruses were used. For this, grapevine plants detected positive for GLRaV-3, GLRaV-4, GVA, and GVB were used. Crude plant extracts from all of these plants along with GGVA-positive and -negative plants were added to the reaction mixtures to ascertain the specificity of the GGVA RPA primers.

### Application of the developed crude sap-based RPA assay for determining the occurrence of grapevine geminivirus A (GGVA) in different grapevine cultivars

Grapevine plant samples collected from different locations from Andhra Pradesh, Maharashtra, and IARI, New Delhi, were used for field validation of the RPA assay and to know the occurrence of GGVA (**Supplementary Table S1**). The standardized crude sap-based RPA assay was performed using all of these collected symptomatic and asymptomatic plants. The standard conventional PCR was also performed parallelly to identify any discrepancies in the detection of GGVA through these two techniques. Crude sap (lysed in 0.5 M NaOH) and DNA extracted from all of these samples were used as templates in the RPA assay and PCR, respectively.

## Results

### High-throughput sequencing and PCR-based confirmation of grapevine geminivirus A (GGVA)

**Figure 1:**
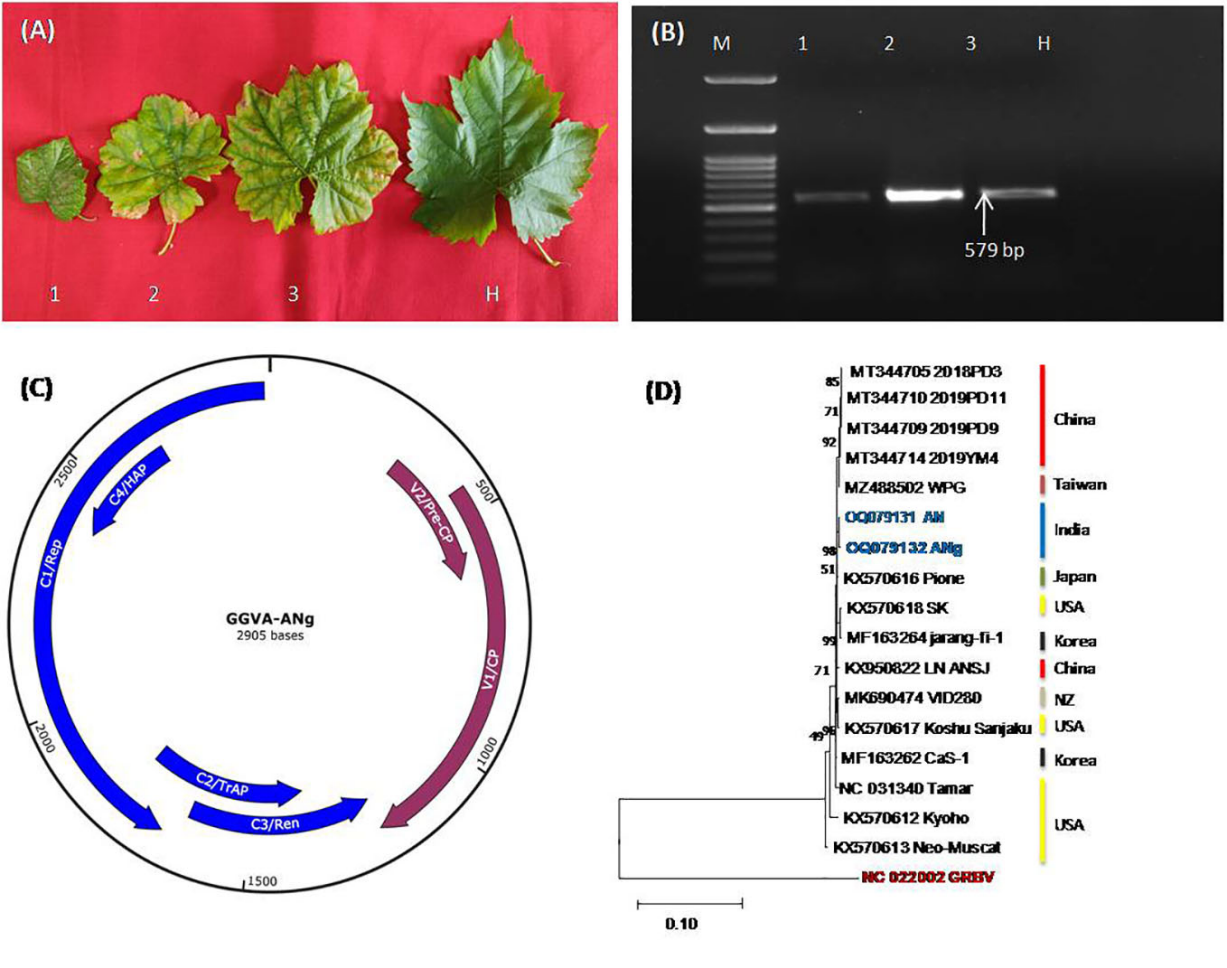
**(A)** Represents symptomatic leaves from three different grapevine cultivars, Super Sonaka, Anushka, and Manik Chaman (Lanes 1, 2, and 3, respectively), and an asymptomatic leaf from Dogridge (Lane H). **(B)** PCR amplicons of 579 bp obtained from amplifications of coat protein genomic region of grapevine geminivirus A (GGVA) using primer pairs of GGVA-Fp and GGVA-Rp synthesized through IDT (Integrated DNA Technologies, Inc., IA, USA). M, 100-bp DNA Ladder; 1, Super Sonaka; 2 Anushka; 3, Manik Chaman plants; H, Healthy plant (Dogridge). **(C)** Represents genomic regions of GGVA isolate ANg (Anushka-grafted plants) as predicted by SnapGene v6.2.1. V1, coat protein (CP); V2, pre-CP; C1, replication-associated protein (Rep); C2, transcriptional activator protein (TrAP); C3, replication enhancer protein (Ren); C4, host activator protein (HAP); intergenic regions (IRs) and a stem-loop structure having a conserved 9-base nucleotide sequence “TAATATTAC” similar to other geminiviruses were also present. **(D)** Phylogenetic relationship between our GGVA isolates AN and ANg with other available world isolates. Phylogenetic analysis is based on maximum likelihood method and Kimura 2-parameter model with 1,000 bootstrap replicates (MEGA11).

Virome analysis of ‘Super Sonaka’ and ‘Anushka’ indicated the presence of GGVA along with the multitude of viruses (unpublished data). These positive samples along with ‘Manik Chaman’ and Dogridge (**Figure 1A**) have been validated through PCR to confirm the HTS results. During PCR, amplicons of 579 bp were observed that indicated the presence of GGVA in ‘Super Sonaka’, ‘Anushka’, and ‘Manik Chaman’, while Dogridge rootstock was found as healthy (**Figure 1B**). This amplified region was cloned in a pGEMT vector, and sequencing was carried out in a paired-end manner at Eurofins Genomics (Bengaluru, Karnataka, India). Sequences obtained showed 98%–100% identity with the corresponding CP region of HTS-recovered genome and other world genomes of GGVA.

The GGVA complete genome (2,905 bp) obtained through HTS from Anushka cultivar was further analyzed for ORF prediction through NCBI ORF Finder [ORF finder Home-NCBI (nih.gov)]. All of the six ORFs, viz., V1, CP (771 nt); V2, pre-CP (312 nt); C1, replication-associated protein (1,212 nt); C2, transcriptional activator protein (420 nt); C3, replication enhancer protein (429 nt); and C4, host activator protein (258 nt), were found in this GGVA genome along with intergenic regions (IRs) and a stem-loop structure having a conserved 9-base nucleotide sequence “TAATATTAC” similar to other geminiviruses (**Figure 1C**). Phylogenetic analysis of the recovered complete genomes with all other NCBI-available world genomes using CLUSTALW program of MEGA11 showed the close relationship of our genomes with all of the available Asian genomes (**Figure 1D**). Furthermore, there was no recombination detected in GGVA genomes as analyzed by the RDP4 package program.

### Validation of RPA primers through conventional PCR

**Figure 2:**
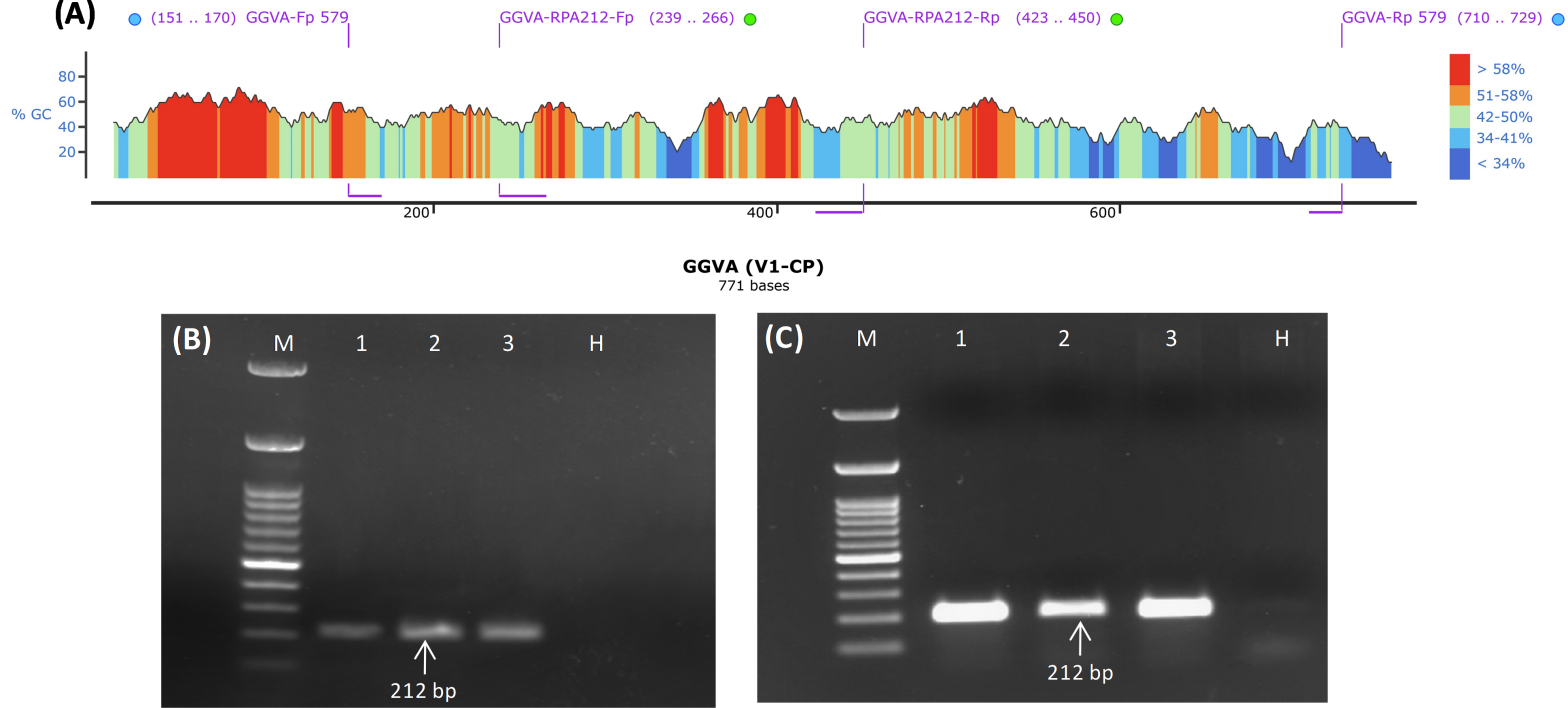
Designing and validation of PCR and RPA-based primers for the detection of GGVA from grapevine plants. **(A)** Diagrammatic illustration of position of PCR and RPA primers within the genomic region (771 bp) of V1/CP gene as visualized by SnapGene v6.2.1 (blue and green dots represent PCR and RPA-based primers, respectively). **(B)** PCR amplicons of 212 bp obtained from amplifications of coat protein genomic region of GGVA using cDNA as template and primer pair of GGVA-RPA212-Fp/Rp synthesized through IDT (Integrated DNA Technologies, Inc., IA, USA). M, 100-bp DNA Ladder; 1, Super Sonaka; 2, Anushka; 3, Manik Chaman plants; H, Healthy plant. **(C)** PCR amplicons of 212 bp obtained from amplifications of coat protein genomic region of GGVA using DNA as template and primer pair of GGVA-RPA212-Fp/Rp synthesized through IDT (Integrated DNA Technologies, Inc., IA, USA). M, 100-bp DNA Ladder; 1, Super Sonaka; 2, Anushka; 3, Manik Chaman plants; H, Healthy plant; GGVA, grapevine geminivirus A; RPA, recombinase polymerase amplification.

GGVA-positive samples (Super Sonaka, Anushka, and Manik Chaman) confirmed through HTS and PCR were used for testing of RPA primers (GGVA-RPA212-Fp and GGVA-RPA212-Rp) designed from the CP genomic region of GGVA. *In silico* hybridization of RPA primer pairs (GGVA-RPA212-Fp and GGVA-RPA212-Rp) and previously tested primer pairs (GGVA-Fp 579 and GGVA-Rp 579) illustrated their position on the CP gene (771 bp) of GGVA (**Figure 2A**). These RPA primers were designed keeping in view the guideline from manufacturers of the RPA kits. Conventional PCR was performed to validate the RPA primers. Amplicons of 212 bp were amplified using cDNA (**Figure 2B**) and DNA (**Figure 2C**) extracted from GGVA-positive samples as a template. These results confirm the functionality of the designed RPA primers that will be used for all of the experiments conducted during the course of the study.

### Crude sap preparation and standardization of the RPA assay

**Figure 3:**
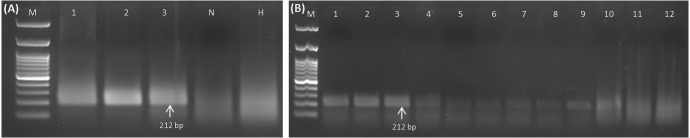
Standardization of RPA assay using RNA and crude sap (isolated from GGVA-positive and healthy grapevine plants) as templates. **(A)** Lane M, 100-bp DNA ladder; 1–3, GGVA-positive plants; N, water control; H, healthy plants. **(A)** RPA-based detection of GGVA from positive samples. Amplicons of 212 bp (primer pairs of GGVA-RPA212-Fp and GGVA-RPA212-Rp) obtained as a result of RNA as template in addition to reverse transcriptase enzyme. **(B)** Comparative analysis of performance of different extraction buffers for the preparation of the crude plant extract. M, 100-bp DNA Ladder; 1–3, 0.5 M NaOH (1:10 w/v); 4–6, phosphate buffer pH 7.4; 7–9, NaOH : EDTA (1:1); 10–12, lysis buffer from DNeasy^®^ Plant Mini Kit (QIAGEN GmbH, Hilden, Germany). GGVA, grapevine geminivirus A; RPA, recombinase polymerase amplification.

Crude extracts prepared using different buffers along the purified RNA and DNA were employed as a template for further validation of the RPA primers (GGVA-RPA212-Fp and GGVA-RPA212-Rp) using RPA reagents. Template of RNA (1 µl) coupled with reverse transcriptase (1 µl) results in amplification of the 212-bp genomic region of CP of GGVA in all of the three previously confirmed positive samples, while amplicons were not obtained in water control and healthy plants (**Figure 3A**). Results obtained by using different crude extracts show variation in amplification, as amplicons obtained from 0.5 M NaOH-based crude extracts were clear and bright as compared to the other three buffers (**Figure 3B**). However, lysis buffer from DNeasy^®^ Plant Mini Kit-based crude plant extract did not result in any amplification. Results from DNA and crude sap (lysed in 0.5 M NaOH) were analyzed on 1.5% agarose gel with and without the addition of 5% SDS (**Supplementary Figures S1A–S1D**). These comparative results showed a clearer and brighter band in the case of the addition of 5% SDS at the time of product loading, whereas the bands of untreated ones were slightly faint. Keeping this in view, the results of all of the downstream processing were obtained using 0.5 M NaOH for crude plant extract preparation.

### Standardization of the incubation temperature and time period for the RPA assay using crude plant extracts

**Figure 4:**
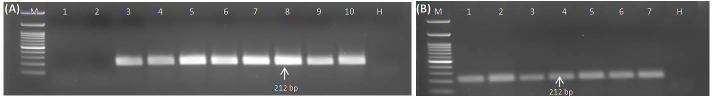
Optimization of the RPA assay to ascertain the best incubation temperature and time using crude extract (isolated from GGVA-positive and healthy grapevine plants) as templates. **(A)** RPA assay performed at different temperatures and a constant time period of 30 min. Lane M, 100-bp DNA ladder; 1, 10°C; 2, 14°C; 3, 18°C; 4, 22°C; 5, 26°C; 6, 30°C; 7, 34°C; 8, 38°C; 9, 42°C; 10, 46°C; H, healthy sample. **(B)** RPA assay performed for different time periods at an isothermal temperature of 38°C. Lane M, 100-bp DNA ladder; 1, 10 min; 2, 15 min; 3, 20 min; 4, 25 min; 5, 30 min; 6, 35 min; 7, 40 min; H, Healthy sample; GGVA, grapevine geminivirus A; RPA, recombinase polymerase amplification.

Crude plant extracts obtained from GGVA-positive samples lysed in 0.5 M NaOH were used as a template to establish the best temperature and period to perform the RPA assay. The RPA assay using crude plant extract was performed at different temperature ranges from 10°C to 46°C at a constant period of 30 min. Results showed that temperature treatment of 26°C, 30°C, 34°C, and 38°C yielded the best amplification of the GGVA genomic region, while the band intensity of amplification was relatively lower at 18°C, 22°C, 42°C, and 46°C as visualized on 1.5% agarose gel (**Figure 4A**). RPA at 10°C and 14°C did not yield any amplification, as these low temperatures may have caused alterations in RPA reactions. Similarly, the RPA assay using crude plant extract was incubated for different periods of 10, 15, 20, 25, 30, 35, and 40 min while keeping an isothermal temperature of 38°C. Results indicated that the reaction starts as early as 10 min of incubation, and this can be visualized clearly on 1.5% agarose gel (**Figure 4B**). However, we chose to perform all of the downstream RPA assays at 38°C for 30 min of incubation, as this has been suggested to be the best by the manufacturers and also by many researchers in previous studies.

### Comparative sensitivity analysis of the RPA assay with conventional PCR using different templates

**Figure 5:**
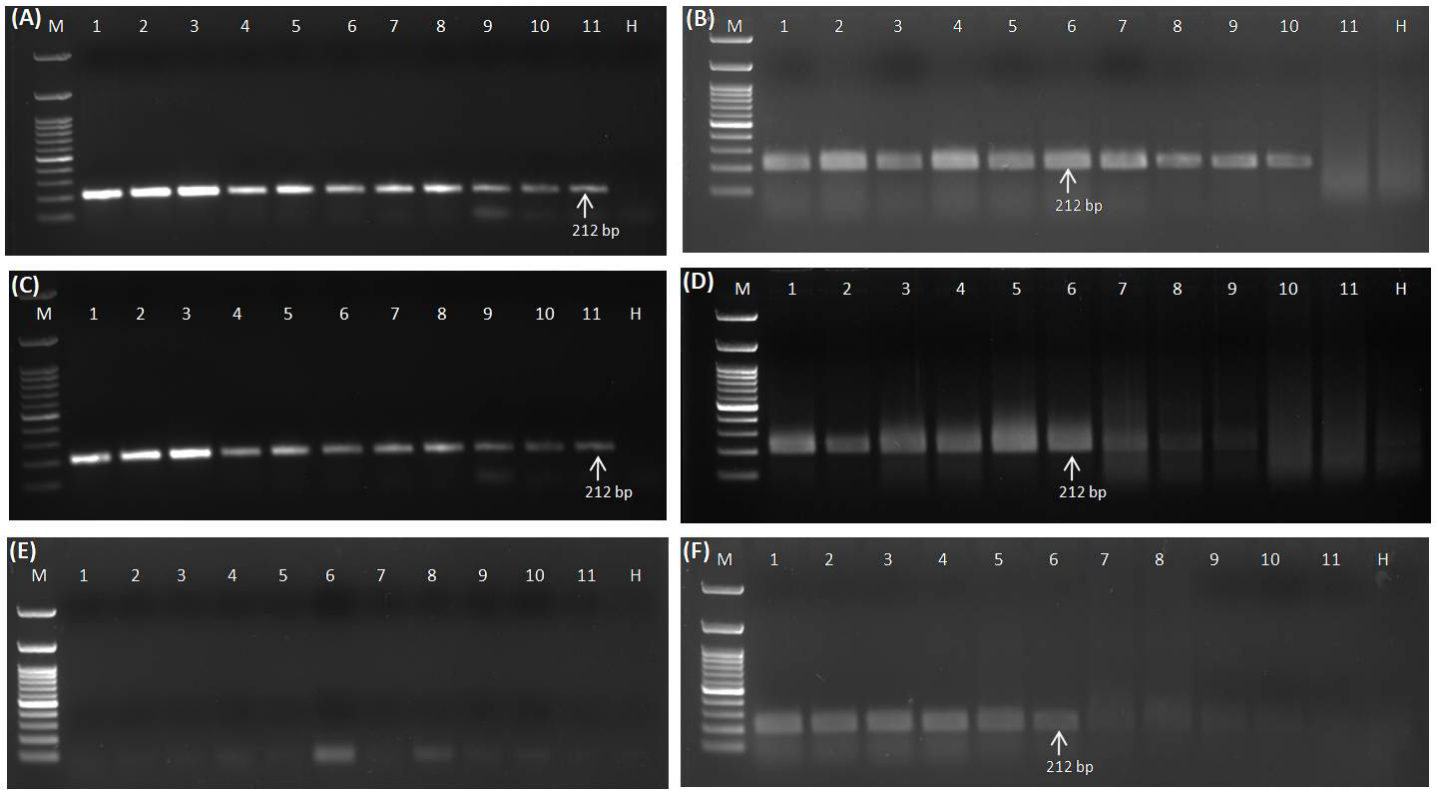
Comparative sensitivity analysis of conventional PCR **(A)** and RPA assay **(B)** to elucidate the detection limit using DNA as template isolated from GGVA-infected plant employing primer pair GGVA-212RPA-Fp/Rp in a 10-fold serial dilution. Lane M, 100-bp DNA ladder; Lane 1, Stock (100 ng); 2, 10^-1^; 3, 10^-2^; 4, 10^-3^; 5, 10^-4^; 6, 10^-5^; 7, 10^-6^; 8, 10^-7^; 9, 10^-8^; 10, 10^-9^; 11, 10^-10^; H, healthy. Comparative sensitivity analysis of conventional PCR **(C)** and RPA assay **(D)** to elucidate the detection limit using cDNA **(C)** and RNA **(D)** as template isolated from GGVA-infected plant employing primer pair GGVA-212RPA-Fp/Rp. In case of RNA template, 1 µl of reverse transcriptase was added in the RPA reaction mixture. Lane M, 100-bp DNA ladder; Lane 1, Stock (100 ng); 2, 10^-1^; 3, 10^-2^; 4, 10^-3^; 5, 10^-4^; 6, 10^-5^; 7, 10^-6^; 8, 10^-7^; 9, 10^-8^; 10, 10^-9^; 11, 10^-10^; H, healthy. Comparative sensitivity analysis of conventional PCR **(E)** and RPA assay **(F)** to elucidate the detection limit using crude plant extract (lysed in 0.5 M NaOH) as template isolated from GGVA-infected plant employing primer pair GGVA-212RPA-Fp/Rp. Lane M, 100-bp DNA ladder; Lane 1, Stock (10^0^); 2, 10^-1^; 3, 10^-2^; 4, 10^-3^; 5, 10^-4^; 6, 10^-5^; 7, 10^-6^; 8, 10^-7^; 9, 10^-8^; 10, 10^-9^; 11, 10^-10^; H, healthy. All PCR reactions were performed at initial denaturation at 94°C for 5 min followed by 35 cycles of denaturation at 94°C for 30 s, annealing at 56°C for 40 s, extension at 72°C for 35 s, and a final extension at 72°C for 10 min. All of the RPA assays were performed at 38°C for 30 min followed by heat inactivation at 65°C for 10 min. GGVA, grapevine geminivirus A; RPA, recombinase polymerase amplification.

Different templates, viz., DNA, RNA, cDNA (prepared from purified RNA), plasmid DNA (GGVA viral insert cloned in pGEMT vector), and crude sap (lysed in 0.5 M NaOH) obtained from positive samples, were serially diluted (10-fold) and used for the analysis of sensitivity for the detection of GGVA through the developed RPA assay and conventional PCR. PCR using isolated DNA as template gave positive amplifications up to 10^-10^ dilutions (equivalent to 0.01 fg µl^−1^) (**Figure 5A**), while RPA using the same templates gave positive amplification up to 10^-9^ dilutions (equivalent to 0.1 fg µl^−1^) (**Figure 5B**). Sensitivity analysis experiment using cDNA (prepared from purified RNA) (template for PCR) and RNA (template for RPA) showed positive amplification up to 10^-10^ (equivalent to 0.01 fg µl^−1^) and 10^-9^ dilutions (equivalent to 1.0 fg µl^−1^), respectively (**Figures 5C, D**). The RPA assay employing crude sap amplified the GGVA CP region up to 10^-9^ dilutions (equivalent to 0.1 fg µl^−1^) (**Figure 5E**), while no amplification was observed in PCR reactions from the same templates (**Figure 5F**). PCR and RPA employing serially plasmid DNA showed positive amplification up to dilutions of 10^-15^ and 10^-10^, respectively (**Supplementary Figures S2A, S2B**). These results showed that the RPA assay is at par with conventional PCR and highly efficient, rapid, and sensitive in the detection of GGVA in grapevine plants.

### Specificity analysis of the RPA assay

RPA reactions employing GGVA-RPA212-Fp/Rp primer pair and crude sap obtained from GGVA-positive and -negative samples along with grapevine samples positive for other viruses, viz., GLRaV-3, GLRaV-4, GVA, and GVB, showed the amplification of GGVA-specific band of 212 bp from the positive sample, while no amplification was observed in all other samples (**Supplementary Figure S3A**). Although GGVA-positive samples (Super Sonaka and Anushka) were also positive for these viruses, they did not show any amplification specific to these viruses and amplified only 212-bp amplicons using GGVA CP-based RPA primer pair (GGVA-RPA212-Fp/Rp). These results showed the specificity of the developed crude sap-based RPA assay for the detection of GGVA. This was further confirmed through cloning of these GGVA-specific amplicons (both PCR and RPA products) and subsequent restriction digestion and sequencing by obtaining 212 bp of sequences having 100% similarity with corresponding CP genomic region and submitted to the NCBI database under accession no. OQ427641 (**Supplementary Figure S3B**). This shows that the developed RPA assay is highly specific for the amplification of GGVA and can be used for detection from field samples.

### Application of the developed crude sap-based RPA assay for determining the occurrence of GGVA in different grapevine cultivars

**Figure 6:**
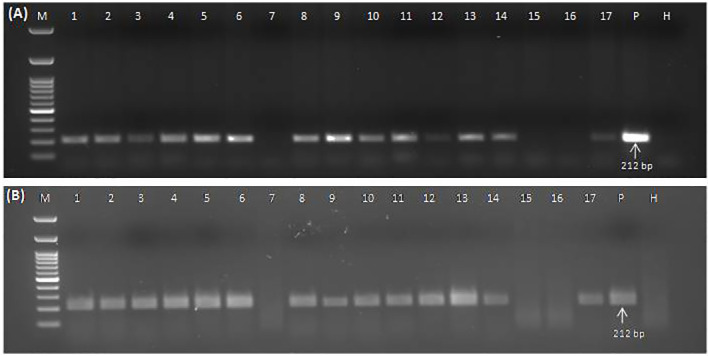
Conventional PCR **(A)** and RPA assay **(B)** to detect GGVA in different field samples using DNA **(A)** and crude plant extract lysed in 0.5 M NaOH **(B)** as template employing primer pair GGVA-212RPA-Fp/Rp. Lane M, 100-bp DNA ladder; Lane 1, Bharat Early (BE); 2, Beauty Seedless (BS1); 3, Beauty Seedless (BS2); 4, Borqui Abyad (BA); 5, Pusa Swarnika (PS1); 6, 1103P; 7, Pusa Navrang (PN1); 8, Pusa Trishar (PT1); 9, Pusa Aditi (PA1); 10, Pusa Aditi (PA2); 11, Pusa Swarnika (PS2); 12, Pusa Swarnika (PS3); 13, Pusa Trishar (PT2); 14, S04; 15, Dogridge (D1); 16, Dogridge (D2); 17, Pusa Trishar (PT3); P, positive control; H, healthy control. PCR reaction was performed at an initial denaturation at 94°C for 5 min followed by 35 cycles of denaturation at 94°C for 30 s, annealing at 56°C for 40 s, extension at 72°C for 35 s, and a final extension at 72°C for 10 min. RPA assays were performed at 38°C for 30 min followed by heat inactivation at 65°C for 10 min. GGVA, grapevine geminivirus A; RPA, recombinase polymerase amplification.

The collected plant samples showed different levels of symptoms ranging from leaf chlorosis, reddening, yellowing, and leaf rolling to no visible symptoms. Detection of GGVA through the conventional PCR (**Figure 6A**) and RPA assay (**Figure 6B**) was performed in parallel for all of these collected samples to evaluate the discrepancies in performance. Results showed that the developed crude sap-based RPA assay is very much effective in the detection of GGVA from grapevine plants. The RPA assay gave a positive reaction for 43 samples out of 60 samples tested (72%) as compared to 39 samples in PCR (65%). Out of these 60 samples, 11 samples were asymptomatic, and three of them showed a positive reaction for GGVA in the PCR, while four showed a positive reaction in the RPA assay. This might be due to the low level of virus titer in asymptomatic plants leading to false-negative results. The remaining samples were symptomatic, and only 13 of them showed a negative reaction for GGVA in the RPA assay. These obtained results demonstrate the feasibility of the crude plant extract-based RPA assay for the detection of GGVA in field samples of grapevine. Test results showed the widespread presence of GGVA in different cultivars across major grapevine areas of India (**Table 1**; **Supplementary Table S1**).

**Table 1:** Validation of the developed crude plant extract-based RPA assay for the detection of GGVA in symptomatic and asymptomatic grapevine plant leaf samples collected from different locations in India.

Sr. No.	Location	Cultivar	Total positive samples/Total samples indexed			
			Purified DNA (PCR)		Crude plant extract (RPA)	
			Symptomatic	Asymptomatic	Symptomatic	Asymptomatic
1.	IARI, New Delhi	Beauty Seedless	3/4	1/2	4/4	2/2
2.		Pusa Trishar	3/3	–	3/3	–
3.		Pusa Swarnika	3/3	–	3/3	–
4.		Pusa Aditi	2/3	–	3/3	–
5.		Pusa Navrang	–	0/2	–	0/2
6.		Bharat Early	1/1	–	1/1	–
7.		Borqui Abyad	1/1	–	1/1	–
8.		1103P	1/1	–	1/1	–
9.		S04	1/1	–	1/1	–
10.		Dogridge	0/2	–	0/2	–
11.	Solapur (Maharashtra)	Manik Chaman	1/1	–	1/1	–
12.		Super Sonaka	2/2	–	2/2	–
13.		Anushka	1/1	–	1/1	–
14.		Dogridge	–	0/3	–	0/3
15.	Anantapur (Andhra Pradesh)	Super Sonaka	6/6	–	6/6	–
16.		Manik Chaman	4/6	–	5/6	–
17.		Dilkush	5/12	2/4	5/12	2/4
18.		Red Globe	1/1	–	1/1	–
19.		Flame Seedless	1/1	–	1/1	–

GGVA, grapevine geminivirus A; RPA, recombinase polymerase amplification.

## Discussion

Grapevine is a host of a large number of RNA and DNA viruses. These viruses were detected and characterized at different timelines using different approaches, viz., ELISA, PCR, and qPCR. Some of the viruses were recently detected in India using HTS (Sidharthan et al., 2020). GGVA was reported from India in the Red Globe cultivar (Sidharthan et al., 2020), although it was not validated through lab-based molecular techniques. Screening of the large number of germplasm materials at the University of California, Davis, showed the presence of the novel GGVA virus in a diverse set of cultivars, source of propagation material that were introduced from different countries like China, Japan, South Korea, and Israel (Al Rwahnih et al., 2017). These results suggested a possible global spread of GGVA that could have escaped the detection procedures due to its novelty and inconsistent symptomatology (Al Rwahnih et al., 2017). Although studies by Sun et al. (2020) associated the leaf edge curling and dwarfism symptoms in *Nicotiana benthamiana* plant with GGVA infection, they could not succeed in establishing a similar association in grapevine plants. These results suggested the possible latent infection or mixed infection of GGVA with other grapevine-infecting viruses. Our results from HTS and bioinformatic analysis also report GGVA infection in Indian grapevine cultivars like Super Sonaka and Anushka in both symptomatic and asymptomatic plants. The complete GGVA genome recovered from Anushka cultivars has a similar genome organization as reported by Sun et al. (2020) representing all of the six ORFs, IRs, and a stem-loop structure having a conserved 9-base nucleotide sequence “TAATATTAC.” The formation of a single clade in the phylogenetic tree and the absence of recombination events indicate the possible origin of this virus from Asian countries, as GGVA isolates reported from US cultivars also had a history from Asian countries only (Al Rwahnih et al., 2017).

Although geminiviruses are known to infect both herbaceous and woody plants of important horticultural crops like citrus, apple, and grapevine (Loconsole et al., 2012; Al Rwahnih et al., 2013; Liang et al., 2015; Al Rwahnih et al., 2017), their detection largely depends on molecular and recently emerging HTS-based techniques that are quite cumbersome in sample preparation and subsequent downstream processing. Once these viruses are detected and characterized, it is important to develop a simplified diagnostic assay for routine detection. In this context, RPA is the recent methodology that has gained the attention of plant pathologists given its simplicity and rapidity in the testing of field and nursery samples for different viruses infecting both field and horticultural crops (Londoño et al., 2016; Kapoor et al., 2017; Romero et al., 2019; Mohandas and Bhat, 2020). RPA coupled with lateral flow dipstick (RPA-LFD) assay was developed for rapid and robust detection of tomato yellow leaf curl virus (TYLCV), although its detection limit was quite low (5 pg µl^−1^) and required laborious DNA extraction protocols (Zhou et al., 2022). Almost similar results were reported by Londoño et al. (2016) for TYLCV detection, and they detected this virus from crude sap itself. Banana bunchy top virus (BBTV) and piper yellow mottle virus (PYMoV) were detected through the crude leaf sap-based RPA assay, but its detection limit was up to 10^-5^ and 10^-3^ dilutions only (Kapoor et al., 2017; Mohandas and Bhat, 2020).

The developed crude sap-based RPA assay was very fast and accurate in the detection of GGVA within 30 min of reaction time, eliminating the requirement of a stringent thermal cycler. Although a LAMP-based detection assay for another ssDNA virus (GRBV) infecting grapevine has been developed by Romero et al. (2019), which required a higher temperature of 65°C, comparatively long incubation period (35 min), and four sets of primers as compared to RPA assay, which can be performed at the temperature range of 18°C–46°C with best amplification at 38°C and needed minimum laboratory setup. Although the developed RPA assay sufficiently amplified the desired amplicons at 18°C and 22°C, its band intensity was relatively lower, as this temperature is not in the most ideal temperature range for recombinase polymerase-based nucleic acid amplification. The developed RPA assay can be completed from reaction to product visualization within a total period of 50–60 min. Although this developed RPA assay requires visualization under gel electrophoresis, it can also be done through the application of probes like RT-exo-RPA (Silva et al., 2015; Srivastava et al., 2019), AmplifyRP^®^ Acceler8™ (Mekuria et al., 2014; Li et al., 2017), and gold nanoparticle (Wang and Yang, 2019). The cost factor for probe-based RPA assays usually limits their application in the routine detection of field samples and planting materials. So keeping in view the limiting factors like the requirement of a stringent thermal cycler, costly reagents, probes, and labor, it is suggested to use crude sap-based RPA assay.

The developed crude sap-based RPA assay is more sensitive and feasible as compared to previously reported detection assays for DNA viruses (Londoño et al., 2016; Kapoor et al., 2017; Mohandas and Bhat, 2020; Zhou et al., 2022) by amplifying the target genomic region. Although the detection limit of crude sap-based RPA was 10 times less as compared to RPA using DNA as a template, this RPA assay does not require lengthy RNA or DNA extraction protocols that are notoriously difficult and time-consuming in the case of crops like grapevine due to the presence of a large number of inhibitors. In the simplified template preparation, only NaOH solution was needed without requiring costly reagents and allows the reaction to start by direct use of this template. This was possibly due to buffering abilities of RPA reagents used and gave an edge over other reported assays that use GEB and GEB3 as extraction buffer and can be used in resource-poor laboratories (Sharma et al., 2023). The present study also reported that RNA isolated for the detection of GGVA can also be used for the RPA-based detection by adding the reverse transcriptase to it. Although the sensitivity of this RNA template-based RPA is comparatively lower (1 fg µl^−1^) than the DNA and crude sap-based RPA assay, it can sufficiently detect GGVA from positive samples. This adds value to our study, as there is no need to isolate RNA and DNA separately for indexing grapevine-infecting RNA and DNA viruses.

The specificity of the developed RPA exists in the amplification of only GGVA-specific amplicons, although tested samples were also positive for other grapevine viruses like GLRaV-3, GLRaV-4, GVA, and GVB. These results were constant with all of the templates (RNA, DNA, and crude sap) used in RPA. This confirms that the developed crude sap-based RPA assay is very specific and robust in the detection of GGVA from infected field samples and did not give any false-positive results. Application of the developed RPA assay presented its effectiveness in the testing of large-scale samples, as 72% of samples collected from different locations were positive in crude sap-based RPA, which was higher as compared to PCR-based results (65% of samples were positive). This might be due to the higher sensitivity of the RPA assay or loss of DNA quality during lengthy extraction protocols for PCR. Furthermore, this study reported widespread occurrence of GGVA infection in Indian grapevine cultivars from different locations including farmer fields. All of these results present the feasibility of crude sap-based RPA assay in the application for large-scale surveillance and planting material certification for GGVA infection.

## Conclusion

In this study, we have successfully characterized the GGVA for the first time in grapevine cultivars from India. Our results indicate the genetic closeness of Indian isolates with other world isolates and suggest that the GGVA infection is prevalent in grapevine plantations in India and may have a significant impact on grape production. Further research is needed to fully understand the impact of GGVA on grapevine growth and yield in India. The developed crude sap-based RPA assay for the detection of GGVA can efficiently detect the virus in field samples with high sensitivity and feasibility. The assay utilizes a crude plant extract and RPA enzymes to amplify and detect GGVA in grapevine samples, making it highly sensitive and specific. The assay eliminates the need for specialized equipment and purification or isolation of the viral DNA, making it a rapid and cost-effective method for the detection of GGVA. The assay has the potential to play a crucial role in the prevention of the spread of GGVA by detecting the presence of the virus in field samples and planting material. Overall, this assay is a valuable tool for the detection and management of GGVA in India and may have implications for other regions where GGVA is prevalent.

## Data availability statement

The datasets presented in this study can be found in online repositories. The names of the repository/repositories and accession number(s) can be found below: NCBI GeneBank database, accession OQ079131 and OQ079132.

## Author contributions

Conceptualization VB, SS. Investigation and Methodology GK, RK, NS, AK. Resources VB. Validation GK, RK, NS, AK. Writing GK. Review and Editing SS, VB, NG. All authors contributed to the article and approved the submitted version.

## Supplementary material

Supplementary Figure 1 Standardization of RPA assay using RNA, DNA and crude sap (isolated from GGVA positive and healthy grapevine plants) as templates. RPA based detection of GGVA from positive samples using DNA **(A)** and crude sap **(B)** as template and products were run on 1.5% gel without adding 5% SDS. RPA based detection of GGVA from positive samples using DNA **(C)** and crude sap **(D)** as template and products were run on 1.5% gel with addition of 5% SDS. Lane M: 100 bp DNA ladder, 1-3: GGVA positive plants, N: Water Control, H: Healthy plants.

Supplementary Figure 2 Comparative sensitivity analysis of conventional PCR **(A)** and RPA assay **(B)** to elucidate the detection limit using plasmid (with GGVA insert) employing primer pair GGVA-212RPA-Fp/Rp in a 10-fold serial dilutions. Lane M: 100 bp DNA ladder, Lane 1: Stock; 2: 10^-1^; 3: 10^-2^; 4: 10^-3^; 5: 10^-4^; 6: 10^-5^; 7: 10^-6^; 8: 10^-7^; 9: 10^-8^; 10: 10^-9^; 11: 10^-10^; 12: 10^-11^; 13: 10^-12^; 14: 10^-13^; 15: 10^-14^; 16: 10^-15^.

Supplementary Figure 3
**(A)** Specificity analysis of crude sap-based RPA assay to elucidate the cross-reaction with other grapevine infecting viruses. Crude sap lysed in 0.5M NaOH was used as template employing primer pair GGVA-212RPA-Fp/Rp. Lane M: 100 bp DNA ladder, Lane 1: GLRaV-3 infected plant sample; 2: GLRaV-4 infected plant sample; 3: GVA infected plant sample; 4: GVB infected plant sample; 5: Negative control; H: healthy sample and P: GGVA positive sample. **(B)** Represents restriction digestion of GGVA specific amplicons (212 bp) from PCR and RPA products cloned in pGEMT vector. Lane M: 100 bp DNA ladder, Lane 1-2: cloned PCR product; 3-4: cloned RPA product and H: negative control.

Supplementary Table 1 List of samples tested for validation of developed crude sap-based RPA assay representing different cultivars and locations of India.
