# Supplementary figures and images for: Development and application of crude sap-based recombinase polymerase amplification assay for the detection and occurrence of grapevine geminivirus A in Indian grapevine cultivars
Source: Front Plant Sci. 2023 Mar 9;14:1151471. doi: 10.3389/fpls.2023.1151471 (PMC10034316; doi:10.3389/fpls.2023.1151471)

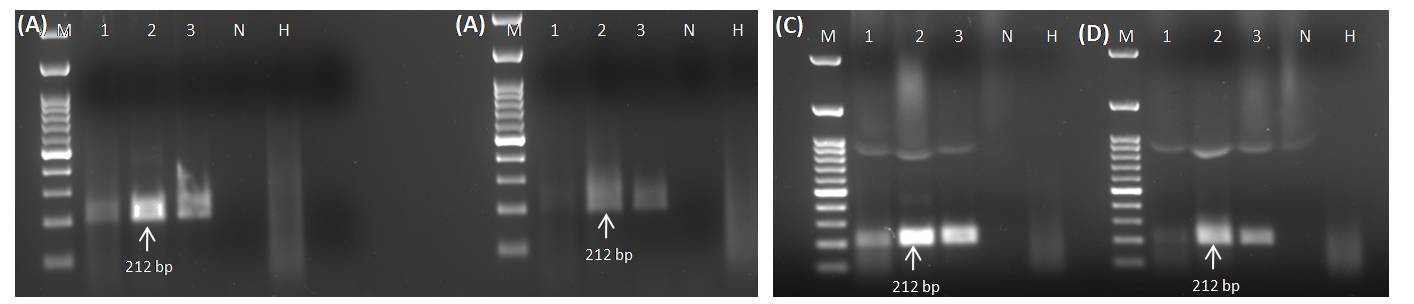

Supplement: Supplementary Figure 1 — Standardization of RPA assay using RNA, DNA and crude sap (isolated from GGVA positive and healthy grapevine plants) as templates. RPA based detection of GGVA from positive samples using DNA (A) and crude sap (B) as template and products were run on 1.5% gel without adding 5% SDS. RPA based detection of GGVA from positive samples using DNA (C) and crude sap (D) as template and products were run on 1.5% gel with addition of 5% SDS. Lane M: 100 bp DNA ladder, 1-3: GGVA positive plants, N: Water Control, H: Healthy plants. [file Image_1.jpeg]

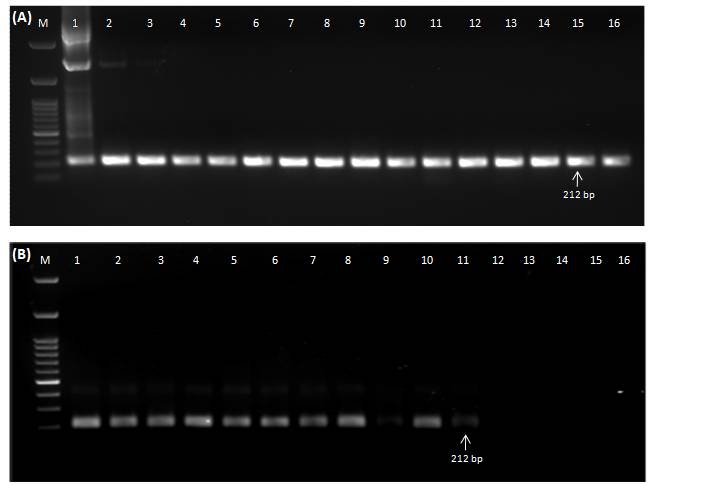

Supplement: Supplementary Figure 2 — Comparative sensitivity analysis of conventional PCR (A) and RPA assay (B) to elucidate the detection limit using plasmid (with GGVA insert) employing primer pair GGVA-212RPA-Fp/Rp in a 10-fold serial dilutions. Lane M: 100 bp DNA ladder, Lane 1: Stock; 2: 10-1; 3: 10-2; 4: 10-3; 5: 10-4; 6: 10-5; 7: 10-6; 8: 10-7; 9: 10-8; 10: 10-9; 11: 10-10; 12: 10-11; 13: 10-12; 14: 10-13; 15: 10-14; 16: 10-15. [file Image_2.jpeg]

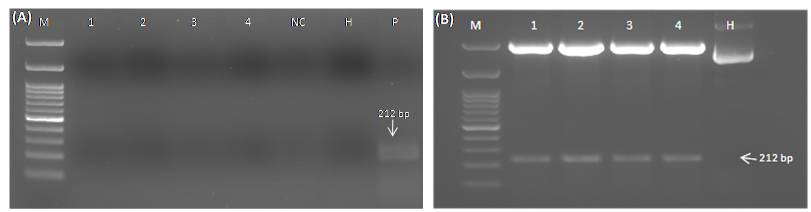

Supplement: Supplementary Figure 3 — (A) Specificity analysis of crude sap-based RPA assay to elucidate the cross-reaction with other grapevine infecting viruses. Crude sap lysed in 0.5M NaOH was used as template employing primer pair GGVA-212RPA-Fp/Rp. Lane M: 100 bp DNA ladder, Lane 1: GLRaV-3 infected plant sample; 2: GLRaV-4 infected plant sample; 3: GVA infected plant sample; 4: GVB infected plant sample; 5: Negative control; H: healthy sample and P: GGVA positive sample. (B) Represents restriction digestion of GGVA specific amplicons (212 bp) from PCR and RPA products cloned in pGEMT vector. Lane M: 100 bp DNA ladder, Lane 1-2: cloned PCR product; 3-4: cloned RPA product and H: negative control. [file Image_3.jpeg]
