# Supplementary material for: Development and application of crude sap-based recombinase polymerase amplification assay for the detection and occurrence of grapevine geminivirus A in Indian grapevine cultivars
Source: Front Plant Sci. 2023 Mar 9;14:1151471. doi: 10.3389/fpls.2023.1151471 (PMC10034316; doi:10.3389/fpls.2023.1151471)
Supplement: Supplementary Table 1 — List of samples tested for validation of developed crude sap-based RPA assay representing different cultivars and locations of India. [file Table_1.docx]

Table 1: List of samples tested for validation of developed crude plant extract based RPA assay representing different cultivars and loations of India

| Sr. No. | District & State | Location | Cultivar | Symptoms | Sample | **GGVA detection** | |
| --- | --- | --- | --- | --- | --- | --- | --- |
|  |  |  |  |  |  | **PCR** | **RPA** |
| 1. | Anantapur (Andhra Pradesh) | Gondireddi  Palli | Manik Chaman | Leaf Crinkling, leaf deformation | MC1 | **+** | **+** |
| 2. |  | Gondireddi  Palli | Super Sonaka | Leaf deformation, smaller crinkled leaves | SS1 | **+** | **+** |
| 3. |  | Gondireddi  Palli | Super Sonaka | Leaf deformation, smaller crinkled leaves | SS2 | **+** | **+** |
| 4. |  | Gondireddi  Palli | Dilkush | No Peculiar symptons | DK1 | **+** | **+** |
| 5. |  | Gondireddi  Palli | Super Sonaka | Leaf Puckering, Leaf rolling | SS3 | **+** | **+** |
| 6. |  | Ramanepalli | Dilkush | Leaf crinkling | DK2 | **+** | **+** |
| 7. |  | Rapthadu,  Anatapur | Manik Chaman | Smaller deformed leaves | MC2 | **-** | **+** |
| 8. |  | Rapthadu | Super Sonaka | Necrosis, Leaf deformation | SS4 | **+** | **+** |
| 9. |  | Rapthadu | Red Globe | Necrosis, Leaf rolling | RG | **+** | **+** |
| 10. |  | Rapthadu | Flame Seedless | Yellowing, Leaf rolling | FS | **+** | **+** |
| 11. |  | Rapthadu | Super Sonaka | Leaf crinkling, discoloration | SS5 | **+** | **+** |
| 12. |  | Rapthadu | Manik Chaman | Small cup shaped and crinkled leaves | MC3 | **+** | **+** |
| 13. |  | Pulakunta | Dilkush | No peculiar symptoms | DK3 | **-** | **-** |
| 14. |  | Hampapuram | Dilkush | No peculiar symptoms | DK4 | **+** | **+** |
| 15. |  | Gollapalli, Anantapur | Manik Chaman | Leaf deformation | MC4 | **+** | **+** |
| 16. |  | Gollapalli, Anantapur | Manik Chaman | Leaf crinkling, deformation | MC5 | **-** | **-** |
| 17. |  | Hampapuram, Anantapur | Dilkush | Leaf deformation | DK5 | **-** |  |
| 18. |  | Uppanesinapalli, Marror, Dharmavaram | Dilkush | Leaf distortion, puckering and crinkling | DK6 | **+** | **+** |
| 19. |  | Darsimala, Dharmavaram | Dilkush | Leaf crinkling | DK7 | **-** | **-** |
| 20. |  | Nadimigadda Palli , Dharmavaram | Dilkush | Leaf puckering and leaf rolling | DK8 | **+** | **+** |
| 21. |  | Mukthapuram, Dharmavaram | Dilkush | No peculiar symptoms | DK9 | **-** | **-** |
| 22. |  | Nadimigadda Palli | Dilkush | Leaf puckering and deformation | DK10 | **+** | **+** |
| 23. |  | Nadimigadda Palli | Dilkush | Leaf crinkling | DK11 | **-** | **-** |
| 24. |  | Nadimigadda Palli | Dilkush | Leaf deformation | DK12 | **-** | **-** |
| 25. |  | Nadimigadda Palli | Dilkush | Leaf crinkling | DK13 | **+** | **+** |
| 26. |  | Darsimala | Dilkush | Leaf crinkling, puckering and deformation | DK14 | **-** | **-** |
| 27. |  | Darsimala | Dilkush | Leaf deformation | DK15 | **-** | **-** |
| 28. |  | Nadimigadda Palli | Super Sonaka | Leaf rolling, crinkling and deformation | SS6 | **+** | **+** |
| 29. |  | Darsimala | Manik Chaman | Leaf crinkling | MC6 | **+** | **+** |
| 30. |  | Ramanepalli | Dilkush | Puckering of smaller leaves | DK16 | **+** | **+** |
| 31. | New Delhi (India) | IARI, New Delhi | Bharat Early | Leaf yellowing, reddening and chlorosis | BE | **+** | **+** |
| 32. |  | IARI, New Delhi | Beauty Seedless | Leaf yellowing and chlorosis | BS1 | **+** | **+** |
| 33. |  | IARI, New Delhi | Beauty Seedless | Leaf yellowing, reddening and chlorosis | BS2 | **+** | **+** |
| 34. |  | IARI, New Delhi | Borqui Abyad | No peculiar symptoms | BA | **+** | **+** |
| 35. |  | IARI, New Delhi | Pusa Swarnika | Leaf yellowing and chlorosis | PS1 | **+** | **+** |
| 36. |  | IARI, New Delhi | 1103P | Leaf yellowing and chlorosis | 1103P | **+** | **+** |
| 37. |  | IARI, New Delhi | Pusa Navrang | No peculiar symptoms | PN1 | **-** | **-** |
| 38. |  | IARI, New Delhi | Pusa Trishar | Leaf yellowing, reddening and chlorosis | PT1 | **+** | **+** |
| 39. |  | IARI, New Delhi | Pusa Aditi | Leaf yellowing, reddening and chlorosis | PA1 | **+** | **+** |
| 40. |  | IARI, New Delhi | Pusa Aditi | Leaf yellowing, reddening and chlorosis | PA2 | **+** | **+** |
| 41. |  | IARI, New Delhi | Pusa Swarnika | Leaf yellowing, reddening and chlorosis | PS2 | **+** | **+** |
| 42. |  | IARI, New Delhi | Pusa Swarnika | Leaf yellowing, reddening and chlorosis | PS3 | **+** | **+** |
| 43. |  | IARI, New Delhi | Pusa Trishar | Leaf yellowing, reddening and chlorosis | PT2 | **+** | **+** |
| 44. |  | IARI, New Delhi | S04 | Leaf yellowing and chlorosis | S04 | **+** | **+** |
| 45. |  | IARI, New Delhi | Dogridge | No peculiar symptoms | D1 | **-** | **-** |
| 46. |  | IARI, New Delhi | Dogridge | No peculiar symptoms | D2 | **-** | **-** |
| 47. |  | IARI, New Delhi | Pusa Trishar | Leaf yellowing, reddening and chlorosis | PT3 | **+** | **+** |
| 48. |  | IARI, New Delhi | Pusa Navrang | No peculiar symptoms | PN2 | **-** | **-** |
| 49. |  | IARI, New Delhi | Beauty Seedless | Leaf yellowing and chlorosis | BS3 | **+** | **+** |
| 50. |  | IARI, New Delhi | Beauty Seedless | Leaf yellowing and chlorosis | BS4 | **+** | **+** |
| 51. |  | IARI, New Delhi | Beauty Seedless | No peculiar symptoms | BS5 | **-** | **+** |
| 52. |  | IARI, New Delhi | Beauty Seedless | No peculiar symptoms | BS6 | **+** | **+** |
| 53. |  | IARI, New Delhi | Pusa Aditi | Leaf yellowing, reddening and chlorosis | PA2 | **-** | **+** |
| 54. | Solapur (Maharashtra) | Nannaj | Super Sonaka | Leaf yellowing and chlorosis | SS | **+** | **+** |
| 55. |  | Nannaj | Anushka | Leaf yellowing, reddening and chlorosis | AN | **+** | **+** |
| 56. |  | Nannaj | Manik Chaman | Leaf yellowing and chlorosis | MC | **+** | **+** |
| 57. |  | Nannaj | Super Sonaka | No peculiar symptoms | SSg | **+** | **+** |
| 58. |  | Nannaj | Dogridge | No peculiar symptoms | D3 | **-** | **-** |
| 59. |  | Nannaj | Dogridge | No peculiar symptoms | D4 | **-** | **-** |
| 60. |  | Nannaj | Dogridge | No peculiar symptoms | D5 | **-** | **-** |
